# Treatment of hidradenitis suppurativa resolves associated hematologic abnormalities

**DOI:** 10.1097/JW9.0000000000000011

**Published:** 2022-03-25

**Authors:** Peyton C. Morss-Walton, Charlotte Greif, Zachary E. Holcomb, Prerna Salian, Tuyen Yankama, Diana Kim, Martina L., Porter, Alexa B. Kimball

**Affiliations:** Clinical Laboratory for Epidemiology and Applied Research in Skin (CLEARS), Department of Dermatology, Beth Israel Deaconess Medical Center, Boston, Massachusetts, University of Massachusetts Medical School, Worcester, Massachusetts; Clinical Laboratory for Epidemiology and Applied Research in Skin (CLEARS), Department of Dermatology, Beth Israel Deaconess Medical Center, Boston, Massachusetts, University of Texas Southwestern Medical School, Dallas, Texas; Harvard Combined Dermatology Residency Program, Department of Dermatology, Massachusetts General Hospital, Boston, Massachusetts; Clinical Laboratory for Epidemiology and Applied Research in Skin (CLEARS), Department of Dermatology, Beth Israel Deaconess Medical Center, Boston, Massachusetts; Department of Pharmacy, Beth Israel Deaconess Medical Center, Boston, Massachusetts; Clinical Laboratory for Epidemiology and Applied Research in Skin (CLEARS), Department of Dermatology, Beth Israel Deaconess Medical Center, Boston, Massachusetts, Tufts University School of Medicine, Boston, Massachusetts; Clinical Laboratory for Epidemiology and Applied Research in Skin (CLEARS), Department of Dermatology, Beth Israel Deaconess Medical Center, Boston, Massachusetts, Department of Dermatology, Beth Israel Deaconess Medical Center, Boston, Massachusetts

**Keywords:** anemia, hidradenitis suppurativa, immunomodulatory, leukocytosis, thrombocytosis

**What is known about this subject in regard to women and their families?**

- Hidradenitis suppurativa is a chronic, debilitating inflammatory skin condition predominantly affecting women.
- Few studies have characterized laboratory abnormalities in patients with severe hidradenitis suppurativa.
- Laboratory abnormalities in patients with severe hidradenitis suppurativa can prompt unnecessary medical resource utilization due to concern for malignancy or infection.

**What is new from this article as messages for women and their families?**

- Patients with severe hidradenitis suppurativa can have laboratory abnormalities related to the systemic inflammatory nature of their disease.
- Initiation of immunomodulatory therapy can normalize these laboratory abnormalities and improve physician- and patient-reported disease severity over time.
- Knowledge that laboratory abnormalities can be seen in severe hidradenitis suppurativa patients can prevent unnecessary workup for infection or malignancy.

## Dear Editors,

Hidradenitis suppurativa (HS) is an inflammatory skin condition predominantly affecting females in North America that has been found to be associated with anemia and leukocytosis.^1–3^ While thrombocytosis is not recognized as related to HS, it would be expected in a systemic inflammatory state.^4^

Laboratory abnormalities in severe HS patients can prompt unnecessary medical utilization due to concern for malignancy or infection, and we have observed patients undergo bone marrow biopsy unnecessarily. Better characterization of laboratory values can prevent unwarranted concern.

We performed an Institutional Review Board-approved retrospective review of HS patients visiting an academic institution in Boston, MA, from 2015 to 2020. Leukocytosis (white blood cell [WBC] > 10 × 10^9^/L), thrombocytosis (platelet > 400 × 10^9^/L), and anemia (men: hemoglobin [Hb] < 13.0 g/dL, women: Hb < 12.0 g/dL) were considered abnormal. Medications were extrapolated from the joint National Psoriasis Foundation-American Academy of Dermatology guidelines.^5^

**Fig. 1.**
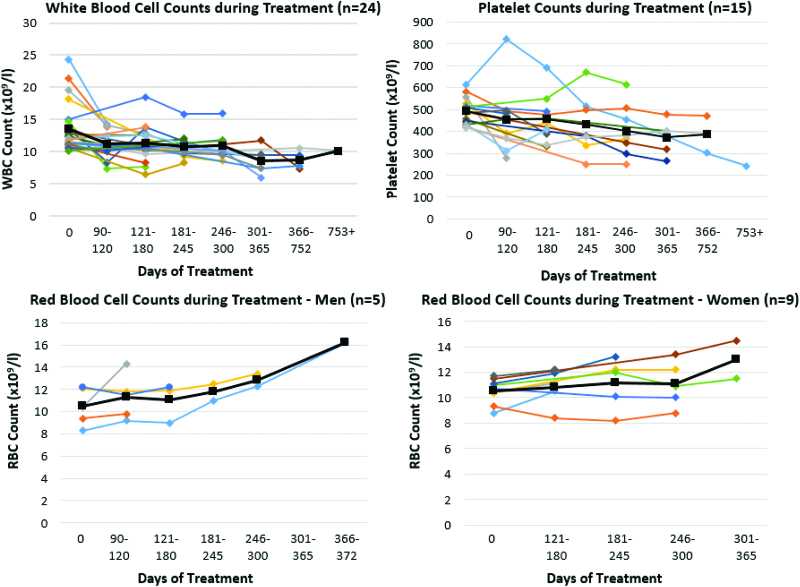
Laboratory abnormalities over time with immunomodulatory therapy. Colored trend lines represent the laboratory values of individual patients. Black lines represent the mean laboratory value for all patients with data in a given time period. RBC, red blood cell; WBC, white blood cell.

Laboratory values were tracked in 3–6-month periods from medication initiation, and patients were consistently on these medications throughout follow-up (Fig. 1). Laboratory values were analyzed for significant changes from therapy initiation. The means ± standard deviations at baseline and follow-up and the mean of individual-level differences were presented. Wilcoxon signed-rank tests were used to determine whether differences between baseline and follow-up laboratory values differed significantly from zero. Tests were 2-sided with nominal significance level of 5%.

We identified thirty patients on immunomodulatory therapy, including methotrexate, adalimumab, infliximab, risankizumab, tofacitinib, or ustekinumab. Seventy-seven percent of patients were female, while 50% were White, 20% Black, 7% Asian, 13% mixed race, and 10% unspecified. Five had coexisting autoimmune disease (Crohn’s disease *n* = 3, Celiac disease *n* = 1, atopic dermatitis *n* = 1). One patient had Hurley I HS, 7 Hurley II, 21 Hurley III, and 1 Hurley II/III. Eighty-three percent of patients (*n* = 25) were on concomitant antibiotics at any point during the study, but patients were on antibiotics for only 13% of all patient days. Eight patients were treated for active infections during the study, and 2 had infections at baseline, 1 with impetigo and 1 with cellulitis.

Twenty-four patients had baseline leukocytosis, which decreased significantly from baseline at 90–120 days (*P* = 0.005) and remained significantly lower than baseline at 181–245 (*P* = 0.012) and 300–365 (*P* = 0.018) days (Table 1; Supplementary Table 1, http://links.lww.com/IJWD/A2). The median follow-up WBC count remained lower than the median baseline WBC count beyond 2 years. WBC levels normalized by 300–365 days. While the 2 patients with infections at baseline were included in the leukocytosis group, they experienced continued decline of WBC levels 6 and 10 months after completing infection treatment.

**Table 1.** Difference in white blood cell, platelet, and hemoglobin levels among patients with abnormal baseline values

Abnormal baseline white blood cell counts				
Time since baseline	Baseline (× 10^9^/L)	Follow-up (× 10^9^/L)	Difference (× 10^9^/L)	*P*
Days 90–120 (*n* = 11)	13.4 (12.0–19.5)	10.8 (9.9–13.8)	–2.6	**0.0051**
Days 121–180 (*n* = 12)	12.4 (11.4–14.1)	11.5 (8.9–13.3)	–0.9	0.13
Days 181–245 (*n* = 10)	13.2 (11.5–15.0)	10.4 (9.7–11.6)	–2.8	**0.012**
Days 245–300 (*n* = 9)	13.4 (10.7–15.0)	10.1 (9.5–11.7)	–3.3	0.11
Days 300–365 (*n* = 7)	12.0 (11.2–13.4)	8.7 (7.4–9.5)	–3.3	**0.018**
Days 365–752 (*n* = 4)	12.5 (11.4–13.5)	8.6 (7.5–10.0)	–3.9	0.068
**Abnormal baseline platelet counts**				
**Time since baseline**	**Baseline (× 10^9^/L**)	**Follow-up (× 10^9^/L**)	**Difference (× 10^9^/L**)	***P***
Days 90–120 (*n* = 9)	508 (431–556)	456 (369–491)	–52	0.17
Days 121–180 (*n* = 10)	499 (431–527)	448 (389–491)	–51	0.11
Days 181–245 (*n* = 7)	511 (419–581)	379 (336–513)	–132	0.13
Days 245–300 (*n* = 8)	501 (433–554)	373 (323–480)	–128	**0.036**
Days 300–365 (*n* = 5)	447 (431–490)	401 (318–401)	–46	**0.043**
Days 365–752 (*n* = 3)	581 (417–613)	391 (302–470)	–190	0.11
**Abnormal baseline hemoglobin—women**				
**Time since baseline**	**Baseline (g/dL**)	**Follow-up (g/dL**)	**Difference (g/dL**)	***P***
Days 90–120 (*n* = 1)	10.4	11.1	+0.7	—
Days 121–180 (*n* = 5)	10.4 (9.3–11.1)	11.1 (10.5–11.9)	+0.7	0.35
Days 181–245 (*n* = 5)	10.7 (10.4–11.0)	12.0 (10.1–12.2)	+1.3	0.35
Days 245–300 (*n* = 5)	10.7 (10.4–11.0)	10.9 (10.0–12.2)	+0.2	0.69
Days 300–365 (*n* = 2)	11.3 (11.0–11.5)	13.0 (11.5–14.5)	+1.7	0.18
Days 365–752 (*n* = 0)	—	—	—	—
**Abnormal baseline hemoglobin—men**				
**Time since baseline**	**Baseline (g/dL**)	**Follow-up (g/dL**)	**Difference (g/dL**)	***P***
Days 90–120 (*n* = 5)	10.4 (9.4–12.1)	11.5 (9.8–11.8)	+1.1	0.35
Days 121–180 (*n* = 3)	12.1 (8.3–12.2)	11.9 (9.0–12.2)	–0.2	0.59
Days 181–245 (*n* = 2)	10.2 (8.3–12.1)	11.8 (11.0–12.5)	+1.6	0.18
Days 245–300 (*n* = 2)	10.2 (8.3–12.1)	12.9 (12.3–13.4)	+2.7	0.18
Days 300–365 (*n* = 0)	—	—	—	—
Days 365–752 (*n* = 1)	8.3	16.2	+7.9	—

Data are shown as median values (with interquartile range also reported). Differences with significant *P* values (≤0.05) are indicated in bold font. “—” refers to a range of numbers. “–” is a minus sign.

Fifteen patients had baseline thrombocytosis (Supplementary Table 1, http://links.lww.com/IJWD/A2). Platelet counts decreased over time, and this difference was significant at 245–300 (*P* = 0.036) and 300–365 (*P* = 0.043) days (Table 1). Platelet counts returned to normal by 365–752 days.

Patients (*n* = 14) with baseline anemia showed nonsignificant improvement in Hb (Table 1; Supplementary Table 1, http://links.lww.com/IJWD/A2). Most patients (57.1%) had resolution of anemia at follow-up (median: 245 days). One patient was treated for anemia during follow-up with ferrous sulfate.

Sixteen of 26 patients (62%) with patient-recorded disease status reported improvement compared with baseline at last laboratory follow-up, while 17 of 25 (68%) had physician-reported improvement.

We observed that immunomodulatory therapy was associated with significant declines in leukocytosis and thrombocytosis with normalization by 300–365 days. This raises awareness that laboratory abnormalities in severe HS patients can reflect underlying disease rather than infection or malignancy.

## Conflicts of interest

The authors made the following disclosures: A.B.K.: Consultant and investigator for AbbVie, Bristol Meyers Squibb, Janssen, Eli Lilly, Novartis, Pfizer, and UCB; consultant for Kymera, Almirall, investigator ChemoCentryx; receives fellowship funding from Janssen and Abbvie; and served as previous Board Of Directors and Past President of the International Psoriasis Council and Board Of Directors of the HS Foundation. M.L.P.: Consultant and investigator for UCB, Pfizer, Eli Lilly, and Novartis and an investigator for Abbvie, Janssen, and Bristol Meyers Squibb. P.C.M.-W., C.G., Z.E.H., T.Y., P.S., and D.K.: None.

## Funding

None.

## Study approval

The author(s) confirm that any aspect of the work covered in this manuscript that has involved human patients has been conducted with the ethical approval of all relevant bodies.

## Patient consent

Informed, written consent was received from all patients and confirmed to the journal prepublication, stating that the patients gave consent for their photos and case history to be published.

## Supplementary materials

Supplementary material associated with this article can be found at http://links.lww.com/IJWD/A2.

## Supplementary Material


