# Supplementary material for: Treatment of hidradenitis suppurativa resolves associated hematologic abnormalities
Source: Int J Womens Dermatol. 2022 Mar 25;8(1):e011. doi: 10.1097/JW9.0000000000000011 (PMC9112385; doi:10.1097/JW9.0000000000000011)
Supplement: Supplementary file 1 [file jw9-8-e011-s001.pdf]

Supplementary Table 1: Demographics of each cohort with baseline laboratory abnormalities

Demographics by Cohort	Abnormal Baseline WBC (n = 24)	Abnormal Baseline Plt (n = 15)	Abnormal Baseline Hb (n = 14)	Abnormal Baseline WBC and Plt (n = 12)	Abnormal Baseline WBC & Hb (n = 10)	Abnormal Baseline Plt and Hb (n = 10)	Abnormal Baseline WBC, Plt, and Hb (n = 8)
**Mean age ± SD (years)**	35.3 ± 14.5	44.5 ± 15.4	36.2 ± 14.6	40.9 ± 16.6	42 ± 16.3	38.6 ± 15.4	33.4 ± 16.4
**Men**	25% (6/24)	27% (4/15)	36% (5/14)	25% (3/12)	40% (4/10)	40% (4/10)	37.5% (3/8)
**Women**	75% (18/24)	73% (11/15)	64% (9/14)	75% (9/12)	60% (6/10)	60% (6/10)	62.5% (5/8)
**White**	54% (13/24)	40% (6/15)	43% (6/14)	50% (6/12)	40% (4/10)	30% (3/10)	37.5% (3/8)
**Hispanic /Latino**	21% (5/24)	13% (2/15)	14% (2/14)	17% (2/12)	20% (2/10)	10% (1/10)	12.5% (1/8)
**Black**	17% (4/24)	40% (6/15)	36% (5/14)	25% (3/12)	30% (3/10)	50% (5/10)	37.5% (3/8)
**Asian**	8% (2/24)	7% (1/15)	7% (1/14)	8% (1/12)	10% (1/10)	10% (1/10)	12.5% (1/8)
**Age of onset (years)**	18 ± 8.2	19 ± 9.3	20 ± 7.6	18 ± 10.3	20 ± 8.6	18 ± 5.5	18 ± 6.6
**Age at Diagnosis (years)**	23 ± 14.4	22 ± 14.4	27 ± 15.7	24 ± 15	36.5 ± 16.5	21.5 ± 15.1	27.5 ± 15.9
**Hurley I**	0	0	7% (1/14)	0	10% (1/10)	0	0
**Hurley II**	25% (6/24)	13% (2/15)	14% (2/14)	17% (2/12)	20% (2/10)	10% (1/10)	12.5% (1/8)
**Hurley III**	75% (18/24)	87% (13/15)	79% (11/14)	83% (10/12)	70% (7/10)	90% (9/10)	87.5% (7/8)
**Mean Baseline HS-PGA**	3.8 ± 0.9	4 ± 0.8	3.9 ± 0.9	4.2 ± 0.8	4 ± 1	4.2 ± 0.7	4.4 ± 0.5

Note: Cohorts with >1 baseline dyscrasia (last four columns) include only patients with all listed dyscrasias, not a subset of the listed dyscrasias. “Platelets” is abbreviated as “Plt”.
